# Laboratory and imaging risk factors for mortality in children with primary hemophagocytic lymphohistiocytosis

**DOI:** 10.3389/fonc.2025.1668762

**Published:** 2025-10-13

**Authors:** Jia Huang, Sipei Xu, Rui Tang, Yan Huang, Wei Li, Chundan Gong, Sijie Gao, Hailun Peng, Li Xiao, Wei Ma

**Affiliations:** ^1^ Department of Radiology, People’s Hospital of Yubei District of Chongqing, Chongqing, China; ^2^ Department of Radiology, the Ministry of Education Key Laboratory of Child Development and Disorders, National Clinical Research Center for Child Health and Disorders, China International Science and Technology Cooperation base of Child development and Critical Disorders, Children’s Hospital of Chongqing Medical University, Chongqing, China; ^3^ The First Medicine College, Chongqing Medical University, Chongqing, China; ^4^ Department of Hematology, Children’s Hospital of Chongqing Medical University, Chongqing, China

**Keywords:** pediatric hematology, primary hemophagocytic lymphohistiocytosis, risk factors, genetic subtypes, organ involvement

## Abstract

**Objectives:**

Primary hemophagocytic lymphohistiocytosis (p-HLH), a genetic disorder characterized by hyperinflammation, is associated with high mortality in pediatric hematology. This study investigates laboratory and imaging risk factors for mortality in p-HLH and its subtypes.

**Methods:**

A retrospective analysis (2012-2024) was conducted on 264 pediatric patients with HLH, categorized into p-HLH and secondary HLH (s-HLH). Five laboratory markers and nine imaging findings were compared between groups and across p-HLH subtypes: familial HLH (F-HLH), immunodeficiency-related HLH (I-HLH), and EBV-driven HLH. Mortality risk factors were analyzed.

**Results:**

The cohort included 264 pediatric patients (median age: 4 years, IQR: 2–7 years, 141 males), with 99 having p-HLH (28 F-HLH, 34 I-HLH, 37 EBV-driven HLH), and 165 having s-HLH (EBV-associated). No significant differences in laboratory parameters were observed between p-HLH and s-HLH. Imaging revealed that p-HLH was associated with less severe ascites, more pronounced hepatomegaly, and greater central nervous system (CNS) involvement than s-HLH. Subgroup analysis showed that F-HLH had more severe CNS involvement, while I-HLH had higher rates of pulmonary complications. Independent mortality risk factors for HLH overall included severe thrombocytopenia (HR = 2.93, 95%CI:1.62-5.30, p < 0.01), CNS involvement (HR = 1.80, 95%CI:1.14-2.84, p = 0.01), and liver/spleen damage (HR = 2.78, 95%CI:1.85-4.18, p < 0.01). For p-HLH, specifically, severe liver/spleen damage (HR = 2.68, 95%CI:1.38-5.21, p < 0.01) and pleural effusion (HR = 3.98, 95%CI:1.20-13.2, p=0.02) were critical factors.

**Conclusion:**

No significant differences in mortality risk were found between p-HLH and s-HLH or among p-HLH subtypes. For p-HLH, severe liver/spleen damage and pleural effusion emerged as key mortality predictors.

## 1 Introduction

Hemophagocytic lymphohistiocytosis (HLH), also known as hemophagocytic syndrome, is a life-threatening condition that predominantly affects pediatric populations. It is marked by hyperactivation of cytotoxic T lymphocytes, natural killer cells, and macrophages, leading to a cytokine storm and multi-organ inflammatory injury (1–3). Primary HLH (p-HLH) is an autosomal or X-linked recessive genetic disorder linked to mutations in genes associated with familial HLH (F-HLH), immunodeficiency-related HLH (I-HLH), and Epstein-Barr virus (EBV)-driven HLH. Secondary HLH (s-HLH), triggered by infections, malignancies, or autoimmune diseases, typically occurs in patients without a family history or identified genetic predisposition; EBV-associated HLH (EBV-HLH) is the most prevalent form (4–7).

While p-HLH and s-HLH share similar clinical manifestations, their underlying causes may lead to distinct outcomes. Prior studies suggest p-HLH is associated with a poorer prognosis, more pronounced organ damage, and higher mortality compared to s-HLH. Nevertheless, these studies often have small sample sizes and focus primarily on clinical biochemical markers, with insufficient consideration of imaging indicators (1, 2, 8–10). Imaging serves as an indispensable tool not only for diagnosing and evaluating the severity of organ involvement but also for elucidating the underlying pathogenesis (11, 12).

This study aims to analyze a large cohort of pediatric patients with HLH, focusing on the investigation of p-HLH and its genetic subtypes, with s-HLH as the control group. Comprehensive collection of key laboratory and imaging data were conducted to identify risk factors associated with mortality in p-HLH and its genetic subtypes.

## 2 Materials and methods

### 2.1 Study population

**Figure 1:**
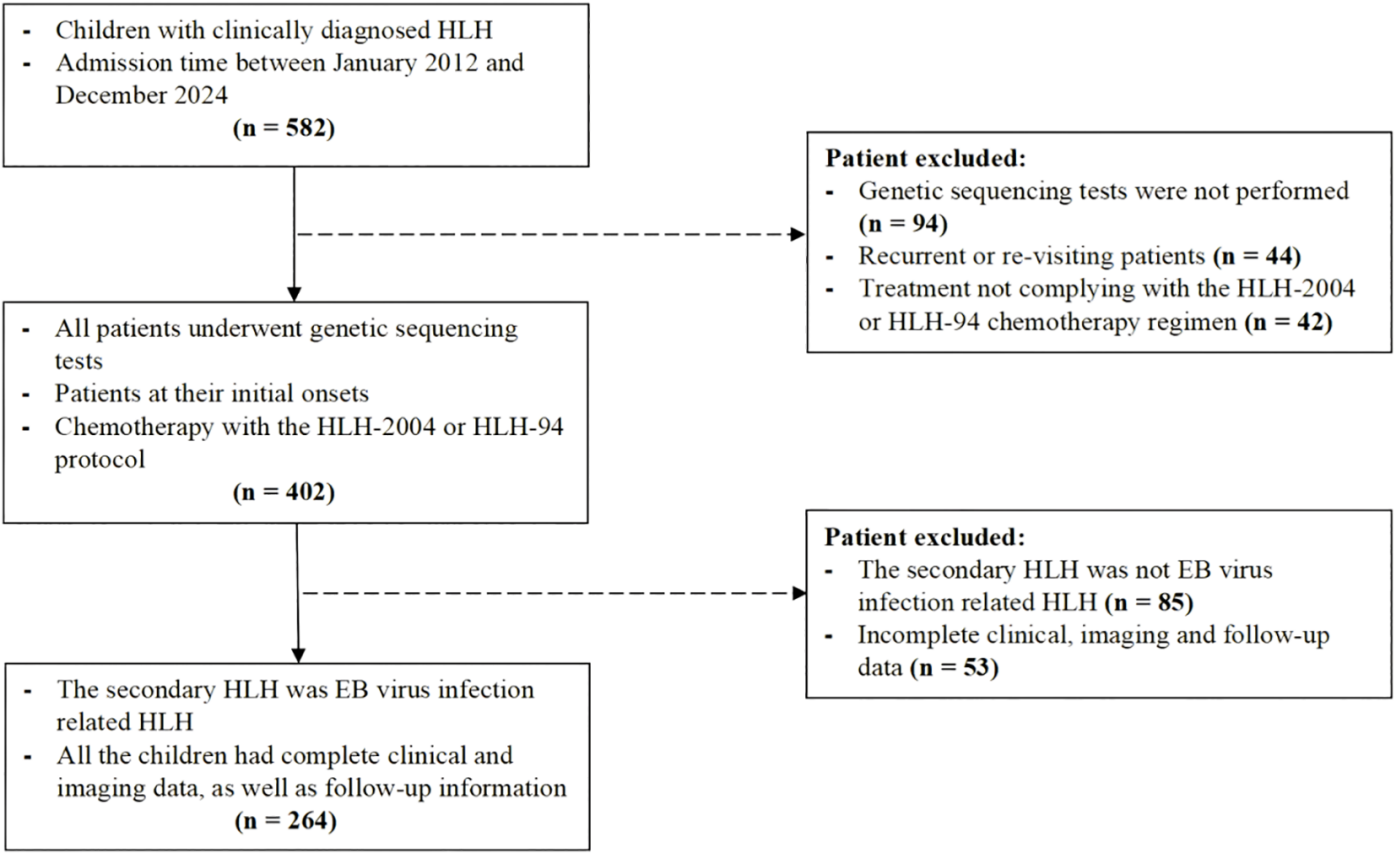
Flowchart of the study population.

This study was approved by the Institutional Review Board (Approval No. 2019-87). Pediatric patients diagnosed with HLH between January 2012 and December 2024 were enrolled. Diagnostic criteria followed the Histiocyte Society guidelines of 2004, requiring fulfillment of both molecular and clinical conditions: 1. Molecular diagnosis: Pathogenic mutations in HLH-associated genes, including F-HLH genes (PRF1, UNC13D, STX11, STXBP2), I-HLH genes (RAB27A, CHS1/LYST, AP3B1), and EBV-driven HLH genes (SH2D1A, BIRC4, MAGT1). 2. Clinical criteria (at least 5 out of 8 items) (1): fever (> 38.5 °C for > 7 days) (2), splenomegaly (3); cytopenia affecting ≥ 2 lineages: hemoglobin < 90 g/L [< 100 g/L for neonates aged < 4 weeks], platelets < 100 × 10^9^/L, neutrophils < 1.0 × 10^9^/L [non-marrow origin]) (4); hypertriglyceridemia (fasting triglycerides > 3 mmol/L or > 3 standard deviations for age) and/or hypofibrinogenemia (< 1.5 g/L or < 3 standard deviations for age) (5); hemophagocytosis in bone marrow, spleen, liver, lymph nodes (6); decreased or absent natural killer (NK) cell activity (7); hyperferritinemia (≥ 500 μg/L); and (8) elevated soluble interleukin-2 receptor (sCD25) (13). Additionally, all included cases must satisfy the following inclusion criteria (1): newly diagnosed cases that were treated in accordance with the HLH-94/2004 protocols (2); for s-HLH controls, only EBV-HLH cases confirmed by positive EBV VCA-IgM and/or serum EBV-DNA levels > 1×10^3^ copies/mL were included to minimize etiological bias (14). The exclusion criteria comprised (1): absence of genetic testing, and (2) incomplete clinical, imaging, or follow-up data (**Figure 1**). Survival status was assessed via telephone or outpatient follow-up until Dec 31, 2024. The endpoint defined as either death or the last follow-up (whichever occurred first), calculated from the date of diagnosis.

### 2.2 Grouping of patients with HLH

Based on clinical diagnostic criteria and the presence of genetic defects (4), the patients with HLH were categorized into two groups: the p-HLH group, which include cases with confirmed genetic defects such as F-HLH, I-HLH, and EBV-driven HLH; and the s-HLH group, which consisted exclusively of the most common EBV-HLH cases (1).

### 2.3 Laboratory data

Clinical data were retrospectively extracted from electronic medical records, including demographic information (gender and age) and five laboratory variables: complete blood count (platelet count [PLT] and hemoglobin [Hb]), liver function marker (lactate dehydrogenase [LDH]), coagulation function (fibrinogen), serum ferritin, and genetic testing results. All laboratory tests and imaging data were collected before initiating the HLH-94/2004 treatment protocol. For indicators measured multiple times during the diagnostic period (such as ferritin and platelet count), this study uniformly used the first measured value for analysis to minimize the influence of treatment intervention on these data.

In this study, genetic testing was primarily performed using analysis based on whole-exome sequencing (WES) and/or targeted sequencing of HLH/immunodeficiency-related gene panels (covering at least 10 or more known HLH-related genes). Inclusion in the p-HLH group required meeting molecular diagnostic criteria: for autosomal recessive HLH-related genes (e.g., PRF1, UNC13D, STX11, STXBP2, RAB27A, AP3B1, etc.), biallelic (homozygous or compound heterozygous) pathogenic/likely pathogenic variants had to be identified; for X-linked genes (e.g., SH2D1A, BIRC4, MAGT1, etc.), pathogenic/likely pathogenic variants consistent with their inheritance pattern had to be detected. All variants were evaluated according to ACMG guidelines. Variants of uncertain significance (VUS) and single heterozygous variants that did not conform to the aforementioned inheritance patterns were excluded from the p-HLH group. Genetic results were classified as either p-HLH (with confirmed pathogenic mutations in HLH-related genes) or non-primary HLH (showing normal sequencing results without definitive mutations or genetic defects).

### 2.4 Imaging data

#### 2.4.1 Equipment and scanning parameters

All imaging data were collected prior to the initiation of the HLH-94/2004 treatment protocol. All patients underwent unenhanced axial brain MRI, as well as axial CT with unenhanced and contrast-enhanced chest and abdominal CT scans. Detailed scanning parameters are provided in **Supplementary Materials 1**.

#### 2.4.2 Image analysis

Images were retrieved from the Picture Archiving and Communication System (PACS) and independently evaluated by two experienced pediatric radiologists (each with > 5 years of experience). Evaluators were blinded to clinical data, and consensus was conducted for any discordant findings. Nine imaging variables were systematically evaluated to assess systemic organ involvement, including CNS involvement, thoracic involvement, and abdominal involvement. Detailed definitions of these imaging variables are provided in **Table 1**.

**Table 1:** A summary of nine imaging variables.

Imaging variables	Diagnostic criteria & clinical implications
CNS Involvement	None: Normal MRI; Mild: Isolated "brain atrophy " changes (early neuroinflammation); Severe: Parenchymal lesions (active neuro-HLH)
Thoracic involvement	
Pulmonary involvement	Mild: < 3 lobes affected (localized infection); Severe: ≥ 3 lobes (diffuse pneumonitis)
Pleural effusion	(Supine position) Mild: pleural effusion depth < 3 cm; Severse: fluid depth ≥ 3 cm (requiring drainage).
Abdominal involvement	
Hepatomegaly	Mild: liver edge above xiphisternal-umbilical midpoint; Severe: below midpoint (portal hypertension, risk of hepatic rupture)
Splenomegaly	Mild: 2 cm below costal margin; Severe: below umbilicus (massive hemophagocytosis)
Hepatic interstitial edema	Periportal "tram-track" sign (inflammatory exudation in hepatic interstitium or lymphatic drainage impairment)
Hepatosplenic enhancement	Heterogeneouls portal-phase enhancement (functional impairment of liver/spleen)
Renal Enhancement	Cortical/medullary hypoenhancement (acute kidney injury)
Ascites	Mild: a crescent-shaped or thin-layered water-like low-attenuation shadow confined to dependent areas; Severe: fluid diffusely distributed around the abdominal organs, and the "intestinal floating sign" may be present.

*HLH*, hemophagocytic Lymphohistiocytosis.

### 3 Statistical analyses

Data were analyzed using SPSS 25.0 (Chicago, IL, USA) and R software (version 4.2.2). Specifically, the following analyses were conducted (1): For intergroup comparisons, the Shapiro-Wilk test was used to evaluated the normality of continuous variables. Normally distributed data were presented as mean ± standard deviation (mean ± SD) and compared using the independent samples t-test. Non-normally distributed variables were expressed as median with interquartile range [M (Q1, Q3)] and analyzed using the Mann-Whitney U test or Wilcoxon rank-sum test. Categorical variables were reported as frequencies (percentages) and assessed using the Chi-square test or Fisher's exact test, as appropriate (2). For survival analysis, mortality was defined as the outcome event, and data were categorized as complete or censored. Kaplan-Meier survival curves were generated, and differences between primary and secondary HLH groups were evaluated using the log-rank test (3). For risk factor analysis, univariate and multivariate Cox proportional hazards regression models were employed to identify potential risk factors associated with mortality in pediatric HLH. Results were expressed as hazard ratios (HRs) and 95% confidence intervals (CIs). A *P*-value < 0.05 was considered statistically significant.

## 4 Results

This retrospective study enrolled 264 pediatric HLH patients (median age 4 years [IQR: 2-7], 141 males) following rigorous screening procedures. The cohort included 99 molecularly confirmed p-HLH cases, comprising 28 familial (with PRF1/UNC13D/STX11/STXBP2 mutations), 34 immunodeficiency-associated cases (with RAB27A/CHS1/AP3B1 mutations), and 37 EBV-driven cases (with SH2D1A/BIRC4/MAGT1 mutations), as well as 165 s-HLH cases associated with EBV infection and negative genetic results. Demographic analysis demonstrated no significant differences in age distribution (p = 0.59) or sex ratio (p = 0.68) between the primary and secondary cohorts (**Table 2**).

**Table 2:** Comparison of laboratory and imaging variables between primary and secondary HLH.

Variables	Primary HLH (n = 99)	Secondary HLH (n = 165)	*P-*value
Demographic data			
Age (year)	4 (3,6)	4 (2,7)	0.59
Sex (case, %)	M, 55 (55.6%) F, 44 (44.4%)	M, 86 (52.1%) F, 79 (47.9%)	0.68
Laboratory variables			
Hemoglobin (g/L)			0.47
* < 90*	10 (10.1%)	10 (6.1%)	
* < 60*	60 (60.6%)	102 (61.8%)	
* < 30*	29 (29.3%)	37 (22.4%)	
Platelet (× 10^9^/L)			0.69
* < 100*	18 (18.2%)	37 (22.4%)	
* < 75*	19 (19.2%)	32 (19.4%)	
* < 50*	62 (62.6%)	96 (58.2%)	
Ferritin (μg/L)			0.38
* < 500*	77 (77.8%)	119 (72.1%)	
* ≥ 500*	22 (22.2%)	46 (27.9%)	
Lactic dehydrogenase (U/L)			0.55
* < 450*	13 (13.1%)	16 (72.1%)	
* < 900*	18 (18.2%)	26 (27.9%)	
* ≥ 900*	68 (68.7%)	123 (74.5%)	
Fibrinogen (g/L)	1.95 (1.52, 3.12)	2.19 (1.53, 3.59)	0.47
Imaging Variables			
CNS involvement (case, %)			0.003*
* None*	24 (24.2%)	74 (44.9%)	
* Mild*	40 (40.4%)	54 (32.7%)	
* Severe*	35 (35.4%)	37 (22.4%)	
Thoracic involvement (case, %)			
Number of the involved lobes			0.44
* < 3 (Mild)*	50 (50.5%)	74 (44.9%)	
* ≥ 3 (Severe)*	49 (49.5%)	91 (55.1%)	
Degree of hydrothorax (case, %)			1.00
* Mild*	15 (15.2%)	26 (15.8%)	
* Severe*	84 (84.8%)	139 (84.2%)	
Abdominal involvement (case, %)			
Hepatomegaly (case, %)			0.03*
* Mild*	43 (43.4%)	95 (57.6%)	
* Severe*	56 (56.6%)	70 (42.4%)	
Splenomegaly (case, %)			0.06
* Mild*	45 (45.4%)	96 (58.2%)	
* Severe*	54 (54.6%)	69 (41.8%)	
HIE (case, %)	42 (42.4%)	86 (52.1%)	0.16
HHSE (case, %)	18 (18.2%)	22 (13.3%)	0.37
HEK (case, %)	4 (4.0%)	14 (8.5%)	0.26
Degree of ascites (case, %)			0.04*
* Mild*	91 (91.9%)	135 (81.8%)	
* Severe*	8 (8.1%)	30 (18.2%)	

*HLH*, hemophagocytic Lymphohistiocytosis; *CNS*, central nervous system; *HIE*, hepatic interstitial edema; *HHSE*, heterogeneous hepatosplenic enhancement; *HEK*, heterogeneous enhancement of kidney. The asterisk (*) indicates a statistically significant differenceBold values and values marked with asterisks (*) indicate statistical significance.

### 4.1 Comparative analysis of primary versus secondary HLH cohorts

For laboratory variables, no statistically significant differences were observed between the two groups (p > 0.05 for all) (**Table 2**).

For imaging variables, significant differences were noted between the two groups regarding the severity of CNS involvement, degree of hepatomegaly, and severity of ascites (all p < 0.05). No significant differences were found in other imaging variables (**Table 2**). Compared to the s-HLH group, the p-HLH group demonstrated more severe CNS involvement (severe involvement: 35.4% vs. 22.4%), more pronounced hepatomegaly (severe hepatomegaly: 56.6% vs. 42.4%), and less ascites (severe ascites: 8.1% vs. 18.2%).

### 4.2 Survival analysis between primary versus secondary HLH cohorts

**Figure 2:**
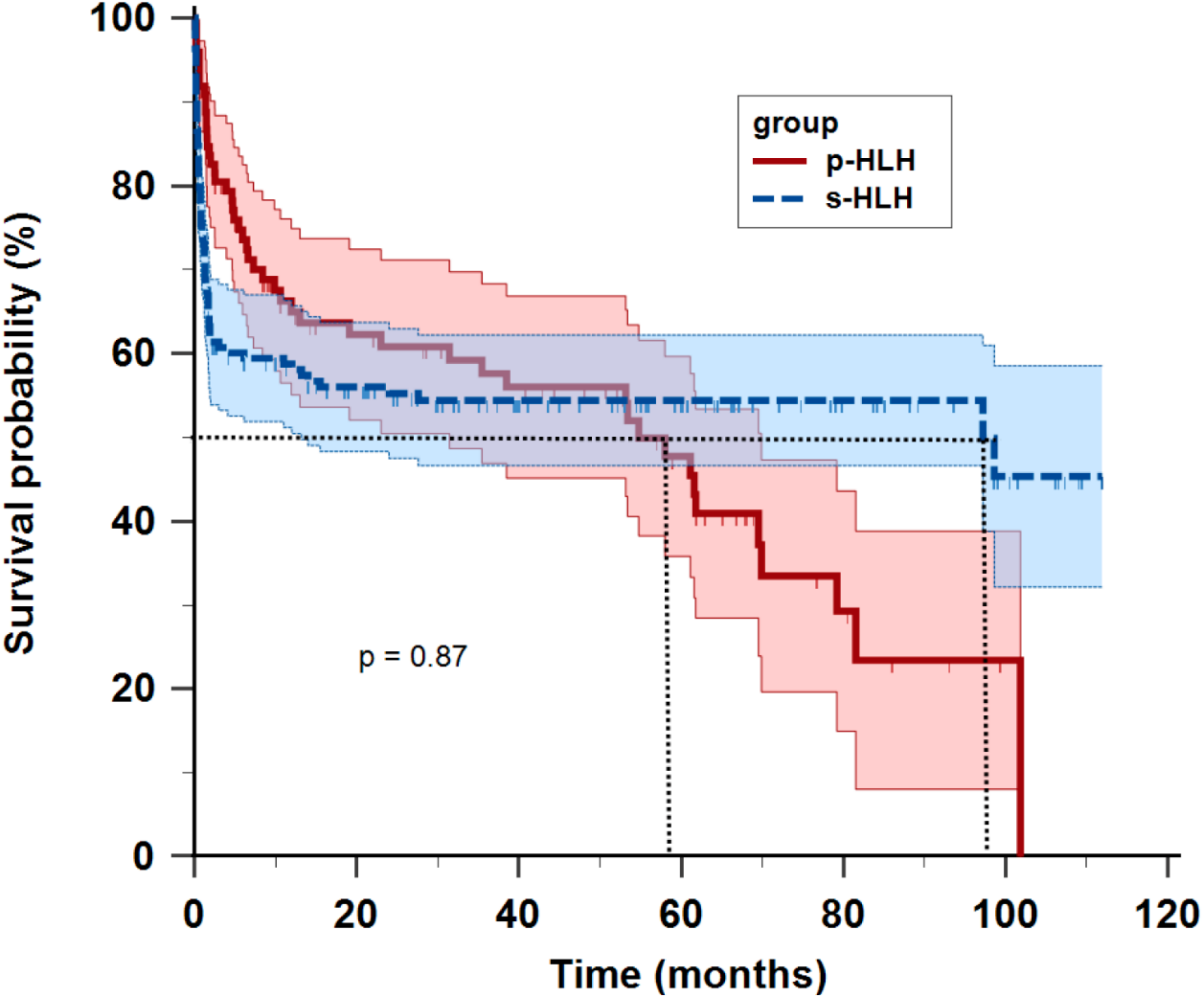
Kaplan-Meier survival curves of children with primary HLH (p-HLH) and secondary HLH (s-HLH). No significant difference was observed between the two groups (p = 0.87).

With a median follow-up duration of 53.2 months (range: 0.1–111 months) as of Dec 31, 2024, the overall median survival time was 61.8 months (range: 0.1-111.9 months). The p-HLH cohort exhibited a mortality rate of 50.5% (50/99) with a 3-year overall survival rate of 56.2% and median survival time of 54.8 months. In contrast, s-HLH cohort demonstrated a mortality rate of 45.5% (75/165), a 3-year overall survival rate of 54.8%, and a median survival time of 97.2 months. Kaplan-Meier analysis indicated no significant difference in overall survival between the HLH groups (HR 1.03, 95%CI 0.72-1.48; p = 0.87) (**Figure 2**).

### 4.3 Analysis of mortality risk factors in HLH children

**Figure 3:**
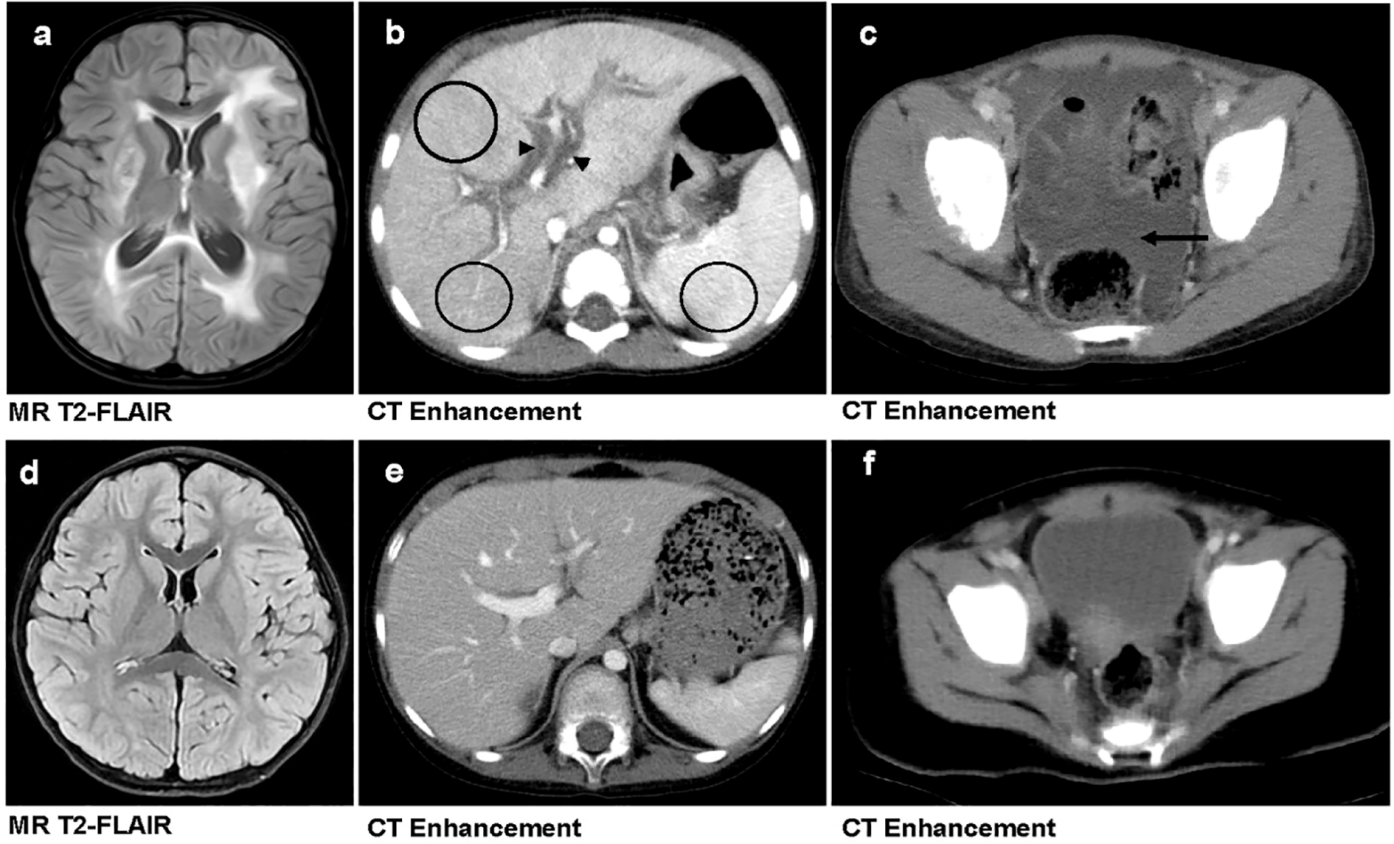
Comparison of imaging findings between primary and secondary HLH in pediatric patients. **(a–c)** A 3-year-old girl with primary HLH (genetic defect: UNC13D, familial HLH type 3 [FHL3]) who succumbed to the disease. Brain MRI reveals widespread bilateral T2-FLAIR hyperintensities, indicative of parenchymal involvement **(a)**. Contrast-enhanced abdominal CT demonstrates hepatic interstitial edema (marked by black triangles) and heterogeneous hepatosplenic enhancement (highlighted by black circles) **(b)**. Contrast-enhanced pelvic CT shows massive ascites (indicated by black arrow) **(c). (d–f)** A 3-year-old boy with secondary HLH who survived. Brain MRI **(d)**, contrast-enhanced abdominal CT **(e)**, and Contrast-enhanced pelvic CT **(f)**—all show no significant abnormalities.

Univariate Cox regression analysis of five laboratory variables and nine imaging variables indicated that severe anemia (Hb<30 g/L), severe thrombocytopenia (PLT<50×10^9^/L), severe CNS involvement, hepatic interstitial edema, heterogeneous liver/spleen enhancement, and massive ascites were significantly associated with poor outcomes in children with HLH (**Table 3**). Multivariate analysis further confirmed that severe thrombocytopenia (HR = 2.93, 95%CI:1.62-5.30, p < 0.01), severe CNS involvement (HR = 1.80, 95%CI:1.14-2.84, p = 0.01), heterogeneous liver/spleen enhancement (HR = 2.78, 95%CI:1.85-4.18, p < 0.01), and hepatic interstitial edema (HR = 1.75, 95%CI:1.22-2.51, p < 0.01) as independent risk factors for HLH-related mortality (**Table 3**). **Figure 3** illustrates the distinct imaging manifestations of these risk factors in two HLH pediatric cases with differing clinical outcomes.

**Table 3:** Analysis of mortality risk factors in pediatric HLH.

Variables	Univariate				Multivariate			
	B	SE	*P*-value	HR (95% CI)	B	SE	*P*-value	HR (95% CI)
Group	0.02	0.18	0.90	1.02 (0.71, 1.47)	0.004	0.19	0.98	1.00 (0.69, 1.46)
Laboratory variables								
*Hemoglobin (< 30 g/L)*	1.38	0.43	< 0.01	3.96 (1.71-9.18)				
*Platelet (< 50 × 10^9/^L)*	1.10	0.29	< 0.01	2.30 (1.71-5.26)	1.07	0.30	< 0.01	2.93 (1.62-5.30)
Imaging variables								
*CNS involvement (severe)*	0.85	0.22	< 0.01	2.34 (1.51-3.61)	0.59	0.23	0.01	1.80 (1.14-2.84)
*HIE*	0.65	0.18	< 0.01	1.92 (1.34-2.75)	0.56	0.18	< 0.01	1.75 (1.22-2.51)
*HHSE*	1.10	0.20	< 0.01	2.30 (2.00-4.47)	1.02	0.21	< 0.01	2.78 (1.85-4.18)
*Ascites (severe)*	0.49	0.23	0.04	1.64 (1.03-2.60)				

*Group:* Refers to HLH classification, i.e., primary HLH and secondary HLH*. HLH*, hemophagocytic lymphohistiocytosis*; CNS*, central nervous system; *HIE*, Hepatic interstitial edema; *HHSE*, heterogeneous hepatosplenic enhancement.

### 4.4 Subgroup analysis of primary HLH genotypes

Based on the defective genes, the p-HLH cases were further classified into three subgroups: F-HLH (n = 28), I-HLH (n = 34), and EBV-driven HLH (n = 37). Potential differences were investigated in laboratory variables, imaging findings, and prognosis among these subgroups. No significant differences were observed among the subgroups regarding sex distribution (p = 0.08) or age at diagnosis (p = 0.12) (**Table 4**).

**Table 4:** Differences in laboratory and imaging variables across the primary HLH subgroups.

Variables	F-HLH (n = 28)	I-HLH (n = 34)	EBV-driven HLH (n = 37)	*p-*value
Demographic data				
Age (year)	4 (2.8,5)	5 (4,7)	4 (2,6)	0.12
Sex (case, %)	M, 13 (46.4%) F, 15 (53.6%)	M, 16 (47.1%) F, 18 (52.9%)	M, 26 (70.3%) F, 11 (29.7%)	0.08
Laboratory variables				
Hemoglobin (g/L)				0.60
* < 90*	4 (14.3%)	4 (11.8%)	2 (5.4%)	
* < 60*	17 (60.7%)	18 (52.9%)	25 (67.6%)	
* < 30*	7 (25.0%)	12 (35.3%)	10 (27.0%)	
Platelet (× 10^9^/L)				0.72
* < 100*	5 (17.9%)	5 (14.7%)	8 (21.6%)	
* < 75*	4 (14.3%)	6 (17.6%)	9 (24.3%)	
* < 50*	19 (67.8%)	23 (67.7%)	20 (54.1%)	
Ferritin (μg/L)				0.33
* < 500*	19 (67.9%)	28 (82.4%)	30 (81.1%)	
* ≥ 500*	9 (32.1%)	6 (17.6%)	7 (18.9%)	
Lactic dehydrogenase (U/L)				0.04*
* < 450*	7 (25.0%)	5 (14.7%)	1 (2.7%)	
* < 900*	7 (25.0%)	4 (11.8%)	7 (18.9%)	
* ≥ 900*	14 (50.0%)	25 (73.5%)	29 (78.4%)	
Fibrinogen (g/L)	2.12 (1.68, 2.97)	1.9 (1.53, 3.64)	1.88 (1.37, 3.12)	0.83
Imaging variables				
CNS involvement (case, %)				0.002*
* None*	3 (10.7%)	7 (20.6%)	14 (37.8%)	
* Mild*	7 (25.0%)	17 (50.0%)	16 (43.2%)	
* Severe*	18 (64.3%)	10 (29.4%)	7 (18.9%)	
Thoracic involvement (case, %)				
Number of the involved lobes				0.02*
> *3*	13 (46.4%)	12 (35.3%)	25 (67.6%)	
* ≥ 4*	15 (53.5%)	22 (64.7%)	12 (32.4%)	
Degree of hydrothorax (case, %)				0.50
* Mild*	6 (15.2%)	5 (14.7%)	4 (14.7%)	
* Severe*	22 (78.6%)	29 (86.3%)	33 (86.3%)	
Abdominal involvement (case, %)				
Hepatomegaly (case, %)				0.75
* Mild*	13 (46.4%)	13 (38.2%)	17 (46.0%)	
* Severe*	15 (53.6%)	21 (61.8%)	20 (54.0%)	
Splenomegaly (case, %)				0.22
* Mild*	11 (39.3%)	13 (38.2%)	21 (56.8%)	
* Severe*	17 (60.7%)	21 (61.8%)	16 (43.2%)	
HIE (case, %)	13 (46.4%)	15 (44.1%)	14 (37.8%)	0.76
HHSE (case, %)	4 (14.3%)	7 (20.6%)	7 (18.9%)	0.81
HEK (case, %)	0 (0%)	2 (5.9%)	2 (5.4)	0.55
Degree of ascites (case, %)				0.16
* Mild*	28 (100.0%)	30 (88.2%)	33 (89.2%)	
* Severe*	0 (0%)	4 (11.8%)	4 (10.8%)	

*HLH*, hemophagocytic Lymphohistiocytosis; *CNS*, central nervous system; *HIE*, hepatic interstitial edema; *HHSE*, heterogeneous hepatosplenic enhancement; *HEK*, heterogeneous enhancement of kidney. The asterisk (*) indicates a statistically significant difference.

Bold values and values marked with asterisks (*) indicate statistical significance.

#### 4.4.1 Comparison of laboratory and imaging findings

Among the p-HLH subgroups, LDH was the only laboratory marker that exhibited a significant difference (p = 0.04; **Table 4**). Patients with F-HLH demonstrated lower LDH levels compared to those in the other two subgroups. No significant differences were observed in the remaining laboratory variables (p > 0.05 for all).

Regarding imaging findings, CNS involvement and pulmonary involvement both showed significant differences across the subgroups (p = 0.002 and p = 0.02, respectively; **Table 4**). The F-HLH subgroup exhibited more severe brain injury compared to the other two subgroups (64.3% vs. 29.4% and 64.3% vs. 18.9%), whereas the I-HLH subgroup demonstrated more pronounced pulmonary damage compared to the other two groups (64.7% vs. 53.5% and 64.7% vs. 32.4%). No significant differences were identified in other imaging features.

#### 4.4.2 Prognostic factors in patients with primary HLH

Univariate Cox proportional hazards modeling revealed that severe Hb reduction, severe CNS involvement, pleural effusion, and heterogeneous hepatosplenic enhancement were significant risk factors for mortality in p-HLH patients (**Table 5**).

**Table 5:** Mortality factors analysis in primary HLH.

Variables	Univariate				Multivariate			
	B	SE	P-value	HR (95% CI)	B	SE	*P*-value	HR (95% CI)
Subtypes of p-HLH								
F-HLH								
I-HLH	0.52	0.36	0.15	1.68 (0.83, 3.37)	0.61	0.39	0.12	1.84 (0.86, 3.91)
EBV-driven HLH	-0.23	0.37	0.53	0.79 (0.39, 1.63)	-0.28	0.41	0.48	0.75 (0.34, 1.66)
Laboratory variables								
Hemoglobin (<30g/L)	1.41	0.62	0.02	4.2 (1.23-13.77)				
Imaging variables								
CNS involvement (severe)	1.05	0.43	0.01	2.86 (1.23-6.64)				
Hydrothorax (severe)	1.38	0.60	0.02	3.99 (1.23-12.93)	1.38	0.61	0.02	3.98 (1.20-13.2)
HHSE	1.05	0.31	< 0.01	2.86 (1.57-5.21)	0.98	0.34	< 0.01	2.68 (1.38-5.21)

*HLH*, hemophagocytic lymphohistiocytosis*; CNS*, central nervous system; *HHSE*, heterogeneous hepatosplenic enhancement; *F-HLH*, Familial HLH; *I-HLH*, Immunodeficiency-related HLH.

Multivariate analysis further demonstrated that heterogeneous hepatosplenic enhancement (HR = 2.68, 95%CI:1.38-5.21, p < 0.01) and pleural effusion (HR = 3.98, 95%CI:1.20-13.2, p = 0.02) were independent prognostic determinants (**Table 5**). Notably, the genetic subtype, which was included as a covariate in the multivariate model, did not significantly influence mortality risk (p > 0.05).

## 5 Discussion

Our comparative analysis of primary and secondary HLH in pediatric patients revealed no statistically significant difference in mortality risk between the two groups, indicating that the presence of genetic defects does not independently affect prognosis. Multivariate analysis identified severe thrombocytopenia (PLT < 50×10^9^/L), severe CNS involvement, heterogeneous hepatic/splenic enhancement, and hepatic interstitial edema as independent risk factors for mortality, reflecting disease severity and multi-organ dysfunction. Furthermore, among primary HLH cases, genetic subtypes showed no significant association with survival outcomes. The key determinants of mortality in primary HLH remained radiologically evident pleural effusion and heterogeneous hepatic/splenic enhancement, underscoring the prognostic value of organ-specific damage. Among these risk factors, severe thrombocytopenia represents the sole clinical parameter, while all others are imaging-based indicators involving the brain, chest, and abdomen. These findings underscore the necessity of conducting comprehensive imaging evaluations of major organs in HLH patients. Furthermore, they provide critical insights for elucidating the role of imaging in the diagnosis and management of HLH, facilitating early detection of significant organ damage and guiding timely intervention to improve organ support and preservation.

Previous studies have indicated that primary HLH is associated with a poorer prognosis compared to secondary HLH. However, the findings of the current study do not corroborate this conclusion. The discrepancy may arise from the fact that in this study, s-HLH was specifically defined as being exclusively EBV infection-associated HLH, thereby excluding cases of HLH caused by other pathogens, tumors, or autoimmune diseases. Furthermore, this study encompassed three subtypes of p-HLH, whereas previous studies typically focused solely on familial HLH and did not account for additional subtypes, which could also contribute to the observed differences in results across studies (2, 8, 10, 15, 16).

Regarding both p- and s- HLH, severe thrombocytopenia represents a significant risk factor for mortality. Specifically, children with severe thrombocytopenia exhibit a 2.93-fold higher mortality risk compared to those with mild thrombocytopenia, a finding that aligns with previous studies (17, 18). Severe thrombocytopenia is closely linked to hemophagocytic activity or the suppression of bone marrow hematopoiesis induced by inflammatory cytokines such as TNF-α and IFN-γ. As the disease progresses, there is a marked reduction in platelet count, which serves as an indicator of disease severity and activity. Additionally, According to previous literature, evidence suggests that severe thrombocytopenia contributes to bleeding in vital organs, with intracranial hemorrhage associated with a mortality rate approaching 100% (18–22). This underscores another critical mechanism linking severe thrombocytopenia to increased patient mortality.

Among the imaging features investigated, severe brain parenchyma lesions, heterogeneous hepatosplenic enhancement, and hepatic interstitial edema were identified as independent risk factors for mortality. Extensive research has consistently shown that CNS involvement serves as a poor prognostic indicator in HLH (18, 22, 23). However, our study revealed that not all patients with CNS involvement have poor outcomes. Mild CNS involvement, characterized by "brain atrophy" changes, was not associated with an increased mortality risk. Conversely, severe CNS involvement, marked by brain parenchyma lesions, demonstrated a strong correlation with elevated mortality risk. Patients with brain parenchyma involvement detected on MRI exhibited a mortality risk 1.8 times higher than those without severe CNS involvement. These findings elucidate the relationship between the extent of CNS involvement and patient mortality more clearly. Therefore, for patients with HLH, we recommend performing early brain MRI if CNS involvement is suspected. However, the CNS symptoms/signs in children with HLH are often clinically atypical and can be easily masked or interfered with by mental status changes and symptoms/signs from other organs. Moreover, the presence of severe coagulopathy, which is a contraindication for lumbar puncture, limits its use for assessment. Therefore, we more strongly recommend that all children with HLH should undergo a brain MRI examination to evaluate for potential CNS involvement. If brain parenchyma lesions are identified, early intrathecal therapy should be initiated to mitigate mortality risk. In cases where only "brain atrophy" changes are observed on MRI, cerebrospinal fluid biochemistry and clinical symptoms should be thoroughly evaluated to assess the severity of involvement, and intrathecal therapy should be considered cautiously to prevent overtreatment (19).

Heterogeneous hepatosplenic enhancement, as well as hepatic interstitial edema, serve as indicators of the degree of hepatic and splenic injury. The heterogeneous enhancement observed in these organs may result from the infiltration of hemophagocytes and lymphocytes, which induces cellular necrosis, dissolution, and abnormal vascularization, thereby contributing to the heterogeneous enhancement of the liver and spleen (24–26). Hepatic interstitial edema further signifies microvascular damage and the severity of inflammation processes (27). In primary HLH, heterogeneous enhancement of the liver and spleen, along with severe pleural effusion, have been identified as independent risk factors for mortality. Elevated levels of inflammatory cytokines enhance capillary permeability, leading to the leakage of fluids and proteins into the pleural cavity, thus causing pleural effusion. The volume of pleural effusion may partially reflect the intensity of inflammation and the degree of vascular compromise (25, 28, 29).

This study has several notable limitations that warrant consideration. First, as a retrospective study, the data collected may be subject to confounding factors and biases inherent in such designs. Second, given the inconsistent treatment protocols for different causes of secondary HLH, particularly in tumor-associated HLH and macrophage activation syndrome, the control group in this study was limited to EBV infection-associated HLH, thus not representing all potential causes of secondary HLH. Third, the sample size for primary HLH remains relatively small, which could potentially compromise the robustness of subgroup analysis based on genetic mutations. Fourth, this study only discussed the impact of one treatment method (the HLH-94 or HLH-2004 chemotherapeutic regimen) on prognosis and did not address the differing outcomes brought by hematopoietic stem cell transplantation. In future research, as the number of children in the cohort accumulates, it will be necessary to introduce risk stratification analysis methods to more accurately identify high-risk populations. Fifth, this study did not directly correlate radiologic evidence of organ damage with clinical evidence of organ dysfunction. For example, the association between severe pulmonary imaging findings and the need for mechanical ventilation was not examined. Similarly, the consistency between imaging findings such as heterogeneous renal enhancement and laboratory indicators of renal dysfunction (e.g., elevated serum creatinine, oliguria, etc.) was not evaluated. This limitation prevents us from determining whether these imaging features directly reflect clinically significant organ dysfunction in patients with HLH. Furthermore, we must acknowledge that the CT scans in this study were not performed using a dual-energy CT (DECT) system. Consequently, non-contrast scans were required prior to contrast administration to obtain baseline attenuation data. Had a DECT system been employed, it could have potentially eliminated the need for a separate non-contrast scan (reducing radiation exposure) and provided richer diagnostic information through material decomposition and virtual monoenergetic imaging applications.

In conclusion, this study demonstrates that while there are variations in certain laboratory and imaging variables between primary and secondary HLH, no significant difference in mortality risk was identified between the two groups. Furthermore, no differences in mortality risk were observed across different genetic subtypes of primary HLH. The critical risk factors for mortality in patients with HLH encompass severe thrombocytopenia, CNS involvement, and liver/spleen damage, and for mortality in patients with primary HLH are severe pleural effusion and liver/spleen damage. Clinicians should not only consider clinical laboratory indicators when evaluating the severity of HLH but also actively incorporate imaging studies to assess the degree of organ damage, thereby facilitating accurate evaluation and timely intervention.

## Data Availability

The original contributions presented in the study are included in the article/**Supplementary Material**. Further inquiries can be directed to the corresponding author.
