# Supplementary material for: Laboratory and imaging risk factors for mortality in children with primary hemophagocytic lymphohistiocytosis
Source: Front Oncol. 2025 Oct 13;15:1668762. doi: 10.3389/fonc.2025.1668762 (PMC12554580; doi:10.3389/fonc.2025.1668762)
Supplement: Supplementary file 1 [file Table1.docx]

**Supplemental Materials 1**

Brain MRI was conducted using either a GE Signa Excite 1.5T scanner (GE, Medical Systems, Milwaukee, WI, USA) or a Philips Achieva 3.0 TX (Philips Healthcare, Best, The Netherlands). Specific scanning parameter details are provided in Supplemental Table 1. All included patients had axial CT with contrast-enhanced chest and abdominal CT images acquired using a LightSpeed VCT 64-slice CT scanner (GE LightSpeed, Waukesha, WI). The acquisition parameters were as follows: tube voltage of 100 kV, tube current of 50 or 100 mAs, slice thickness of 1.0 mm, and slice interval of 1.0 mm. The contrast agent iohexol (300 µg/mL; Jiangsu Hengrui Pharmaceuticals Co. Ltd, Jiangsu, China) was administered intravenously into the antecubital vein via a high-pressure injector (ACIST Medical Systems, Inc, Eden Prairie, MN) at a flow rate of 2 mL/s. Venous phase images were subsequently acquired ~60 s after injection.

**Supplemental Table 1** Detailed parameters for brain MRI scanning

|  | Parameters | 1.5 T MRI system | 3.0 T MRI system |
| --- | --- | --- | --- |
| T1-weighted image | TE/TR (msec) | 15-20/2000-2300 | 20/2000 |
|  | NEX/NSA | NEX = 2 | NSA = 1 |
|  | FOV (cm) | 24 × 24 | 20 × 20 - 22 × 22 |
|  | Matrix size | 320 × 320 | 300 × 240 |
|  | Slice thickness/Slice gap (mm) | 5/1 | 5-6/1 |
| T2-weighted image | TE/TR (msec) | 100-110/3000-3400 | 80/3500 |
|  | NEX/NSA | NEX = 2 | NSA = 1 |
|  | FOV (cm) | 24 × 24 | 20 × 20-22 × 22 |
|  | Matrix size | 320 × 320 | 300 × 240 |
|  | Slice thickness/Slice gap (mm) | 5/1 | 5-6/1 |
| T2 ﬂuid-attenuated inversion recovery (T2-FLAIR) | TE/TR/TI (msec) | 110-130/8000-8500/2100 | 125/7000/2500 |
|  | NEX/ NSA | NEX = 2 | NSA = 2 |
|  | FOV (cm) | 24 × 24 | 20 × 20 - 22 × 22 |
|  | Matrix size | 320×320 | 300×240 |
|  | Slice thickness/Slice gap(mm) | 5/1 | 5-6/1 |
| Diffusion-weighted imaging (DWI) | TE/TR (msec) | 100/5000 | 1780-1960/75-80 |
|  | *b* value (s/mm^2^) | 0, 1000 | 0, 1000 |
|  | NEX/ NSA | NEX = 1 | NSA = 1 |
|  | FOV (cm) | 24 × 24 | 20 × 20-22 × 22 |
|  | Matrix size | 320 × 320 | 300 × 240 |
|  | Slice thickness/Slice gap (mm) | 5/1 | 5-6/1 |
